# Evaluation of Infrared Photoprotective Potential of Cosmetic Ingredients Using Directional-Hemispherical Reflectance in an Ex Vivo Model

**DOI:** 10.3390/ph19081143

**Published:** 2026-07-24

**Authors:** Elżbieta Mickoś, Paula Babczyńska, Magdalena Hartman-Petrycka, Sławomir Wilczyński

**Affiliations:** 1 Department of Basic Biomedical Science, Faculty of Pharmaceutical Sciences in Sosnowiec, Medical University of Silesia, 40-055 Katowice, Poland; d200890@365.sum.edu.pl (E.M.); mhartman@sum.edu.pl (M.H.-P.); swilczynski@sum.edu.pl (S.W.); 2 Doctoral School, Medical University of Silesia, 40-055 Katowice, Poland

**Keywords:** infrared radiation, photoprotection, ferulic acid, pectin, dextran, directional hemispheric reflectance, thermal emissivity

## Abstract

**Background**: Infrared (IR) radiation constitutes the dominant component of the energy reaching the Earth’s surface and plays a significant role in the photochemical and thermal processes occurring in the skin. The aim of this study was to evaluate the photoprotective potential of selected cosmetic ingredients—ferulic acid, citrus pectin, and dextran—against IR radiation using the directional hemispheric reflectance (DHR) method in an ex vivo model. **Methods**: Formulations based on an amphiphilic carrier (Lekobaza) containing various concentrations of the tested substances were developed, and their optical and thermal properties were evaluated. **Results**: The results showed that the application of all formulations led to a statistically significant reduction in reflectance in the near- and mid-infrared range, indicating an increase in the absorption of radiation energy within the formulation layer. The strongest absorption effect was observed for ferulic acid, which—in addition to its antioxidant properties—exhibits the ability to absorb IR energy and dissipate it as heat. Pectin and dextran formed a water-binding hydrocolloid matrix on the surface, acting as a selective “water filter” for long-wavelength radiation (IR-B and IR-C). At the same time, all the tested systems increased the surface’s thermal emissivity, which promotes more efficient dissipation of absorbed energy through radiative cooling and may support the skin’s natural thermoregulation. **Conclusions**: The obtained data indicate that protection against IR radiation should not be defined solely as the physical reflection of radiation, but as a complex process of managing the skin’s energy balance, encompassing surface absorption, heat dissipation, and the neutralization of biological effects. The results support the development of hybrid photoprotective systems combining antioxidant and thermoregulatory mechanisms to prevent thermal aging-related changes.

## 1. Introduction

Infrared (IR) radiation is an important region of the electromagnetic spectrum that is non-ionizing and invisible to the human eye, yet commonly found in both natural and technological environments. Its interaction with matter occurs primarily through the excitation of vibrations and rotations in chemical bonds, particularly O–H, C–H, and N–H, which leads to an increase in the kinetic energy of molecules and manifests macroscopically as a thermal effect. Due to the relatively low energy of photons, IR radiation is incapable of causing ionization or breaking chemical bonds, which distinguishes it from ultraviolet radiation and visible light. According to the principles of quantum mechanics, the energy of a photon decreases with increasing wavelength, which determines the specific biological and physicochemical properties of infrared radiation [1]. The ISO 21348:2007 standard distinguishes three spectral ranges of IR radiation: near, mid, and far infrared, which differ in wavelength and photon energy, and thus in their potential range of interactions with matter [2].

The percentage contribution of individual radiation bands in the solar spectrum at sea level (0 m a.s.l.) varies depending on the source, due to differences in measurement methodologies. Regardless of these differences, ultraviolet radiation (UVR) accounts for the smallest energy share, followed by visible light, while infrared radiation is the dominant component, accounting for approximately 8% for UVR, 42.3% for the visible range, and 49.4% for IR [1]. Current data indicate that visible and infrared radiation, although outside the ultraviolet range, can significantly contribute to skin damage. Due to the dominant share of IR radiation in the solar spectrum and additional sources of exposure, the skin may be exposed to high cumulative doses, which, at high intensities, lead to the development of pathological changes [3]. The penetration depth of infrared radiation in the skin decreases as the wavelength increases. The IR-A range affects all layers of the skin, though it does not cause a significant increase in surface temperature and is absorbed mainly in the epidermis, dermis, and subcutaneous tissue. IR-B radiation also reaches all layers of the skin, whereas IR-C is limited to the epidermis, where it causes a rise in temperature due to intense absorption by water in the superficial layers. In dermatology, a chronic dermatosis known as erythema ab igne has been described, developing as a result of prolonged, repeated exposure of the skin to thermal radiation at an intensity that does not cause burns. Skin lesions include characteristic hyperpigmentation with a brownish-red color, the presence of telangiectasia, epidermal atrophy, dilation of blood vessels, and the deposition of melanin and hemosiderin [4]. The photoaging process induced by infrared radiation exhibits numerous similarities to skin changes caused by ultraviolet radiation. At the same time, studies indicate distinct cytokine profiles and differences in the activated signaling pathways involved in the skin’s biological response to these two types of radiation [5]. Repeated exposure of the skin to high-dose infrared radiation leads to increased activity of matrix metalloproteinase-1 (MMP-1), which results in disruption of tissue homeostasis. This results in increased degradation of extracellular matrix components, including collagen and elastin, while simultaneously weakening the mechanisms that inhibit this process, which depend on tissue inhibitor of metalloproteinase-1 (TIMP-1) [6,7]. At the molecular level, this effect is initiated by the absorption of IR-A photons within complexes of the mitochondrial respiratory chain of dermal fibroblasts, which triggers a retrograde signaling response and the intramitochondrial generation of reactive oxygen species (ROS). This oxidative stimulus activates the mitogen-activated protein kinase (MAPK) cascade—in particular ERK1/2 and p38-MAPK—leading to activation of the transcription factor AP-1 (a c-Fos/c-Jun heterodimer), which up-regulates MMP-1 gene transcription; the concomitant down-regulation of TIMP-1 further shifts the protease–antiprotease balance towards enhanced degradation of type I collagen and elastin. Infrared radiation is associated with thermal radiation. It has been demonstrated that heat increases metalloproteinase activity, modulates the synthesis of elastin and fibrillin, enhances angiogenesis, induces oxidative stress through an increase in reactive oxygen species, and promotes the recruitment of immune cells [6]. It has been demonstrated that exposure of the skin to high-dose rate infrared radiation, as well as to heat, leads to an increase in the number of mast cells and an upregulation of tryptase expression. Furthermore, studies show that a single dose of IR radiation causes an increase in type I procollagen expression, whereas repeated exposure results in its downregulation. A similar pattern of changes has been observed in vivo for transforming growth factor β (TGF-β), a key regulator of fibroblast proliferation and procollagen synthesis—its expression increases after a single exposure to IR, whereas it decreases with prolonged, repeated exposure [8,9].

Currently, strategies for protecting the skin against infrared radiation focus primarily on mitigating the secondary biological effects of exposure rather than physically blocking the radiation. The basis of this protection is the topical application of formulations containing antioxidants, whose role is to neutralize IR-induced oxidative stress. It has been demonstrated that a mixture of antioxidants including vitamin C, vitamin E, ubiquinone, and grape seed extract effectively inhibits IR-A-induced MMP-1 expression, confirming the validity of this approach [5]. At the current stage of knowledge, the application of antioxidants is the only documented strategy for protection against the negative effects of IR radiation, as there are no reports in the literature of cosmetic ingredients capable of forming an IR barrier analogous to the filters used in UVR protection.

Studies evaluating the effectiveness of classic sunscreens in the context of protection against IR radiation provide ambiguous results; however, most of them indicate their limited effectiveness in this regard [7,10,11]. Spectrophotometric analyses have shown that UVR filters do not block radiation in the near- and mid-infrared ranges, and IR transmittance can exceed 50%, in contrast to their significantly higher effectiveness in the UVR range [12]. Similar observations were obtained in in vivo studies based on measurements of the skin’s radiation reflectance [10]. In turn, studies using EPR spectroscopy and Monte Carlo simulations suggest that some formulations containing UVR filters and antioxidants may limit the formation of free radicals following IR exposure and exhibit properties that scatter this radiation, though without the ability to absorb it [13]. Consequently, protection against IR radiation remains a research challenge and requires the development of standardized methods for evaluating the radiation-protective properties of cosmetics in this spectral range.

A systematic increase in human skin exposure to infrared radiation is being observed, which is a consequence of several coexisting factors. First and foremost, IR radiation accounts for more than half of the solar radiation energy reaching the Earth’s surface. Additionally, increased outdoor activity and growing exposure to IR radiation from anthropogenic sources, such as artificial lighting or medical procedures, including esthetic medicine treatments, lead to further intensification of exposure. Paradoxically, the use of sunscreens containing UVR filters also contributes to the phenomenon of excessive IR exposure, as the elimination of acute symptoms of UVR exposure, such as erythema or sunburn, encourages longer periods of sun exposure. As a result, despite protection against UVR, the skin may remain exposed to infrared radiation for a longer period of time [11].

Therefore, the aim of the present study was to evaluate the infrared photoprotective potential of selected cosmetic ingredients, including dextran, pectin, and ferulic acid. Dextran is a microbial polysaccharide [14], whereas pectin is a natural plant polysaccharide widely used as a gelling and rheology-modifying agent [15,16]. Their infrared photoprotective potential was assessed using directional-hemispherical reflectance and thermal emissivity measurements in an ex vivo model.

## 2. Results

### Results of Ex Vivo Tests (On the Material): Reflectance and Thermal Emissivity

In the ex vivo tests conducted on the material, reflectance and thermal emissivity were evaluated. Detailed measurement results are summarized in Table S2. It was demonstrated that directional-hemispherical reflectance in the 700–1100 nm spectral range was statistically significantly reduced after the application of the drug base (B; *p* < 0.001), 5% dextran (D5%; *p* = 0.004), 3% pectin (P3%; *p* = 0.002), 5% pectin (P5%; *p* = 0.002), and ferulic acid at concentrations of 0.5% (FA0.5%; *p* < 0.001) and 1% (FA1%; *p* < 0.001).

In this spectral range, however, no significant changes were observed after the application of 10% dextran (D10%).

In the 1000–1700 nm wavelength range, a statistically significant decrease in directional-hemispheric reflectance was observed after application of the drug base and all tested formulations, including 5% and 10% dextran, 3% pectin, 0.5% and 1% ferulic acid (*p* < 0.001), as well as 5% pectin (*p* = 0.001).

A similar effect was observed in the 1700–2500 nm spectral range, where the application of the drug base and all analyzed preparations (B, D5%, D10%, P3%, P5%, FA0.5%, FA1%) led to a statistically significant reduction in directional-hemispheric reflectance (*p* < 0.001).

In the 700–1100 nm spectral range, the median percentage change in directional-hemispheric reflectance after applying the tested formulations to the test material was 0% for the material alone (C). After application of the preparations, slight decreases in reflectance were observed, reaching −0.4% for the drug base (B), −0.3% for 5% dextran (D5%), −0.2% for 10% dextran (D10%), −0.7% for 3% pectin (P3%), −0.5% for 5% pectin (P5%), and −0.5% and −0.8% for ferulic acid at concentrations of 0.5% (FA0.5%) and 1% (FA1%), respectively.

In the wavelength range of 1000–1700 nm, the median percentage difference in reflectance remained at 0% for the test material, whereas a more pronounced decrease in this parameter was observed after the application of the formulations. The decrease was −5.6% for the drug base, −3.3% for 5% dextran, −5.1% for 10% dextran, −4.0% for 3% pectin, −1.5% for 5% pectin, −6.3% for 0.5% ferulic acid, and −10.1% for 1% ferulic acid.

The greatest changes were observed in the 1700–2500 nm spectral range. The median percentage difference in directional-hemispheric reflectance was 0% for the test material, while after application of the preparations, the decrease in reflectance reached −21.6% for the drug base, −14.2% for 5% dextran, −21.2% for 10% dextran, −17.4% for 3% pectin, −4.6% for 5% pectin, −23.6% for 0.5% ferulic acid, and as much as −33.7% for 1% ferulic acid.

**Figure 1:**
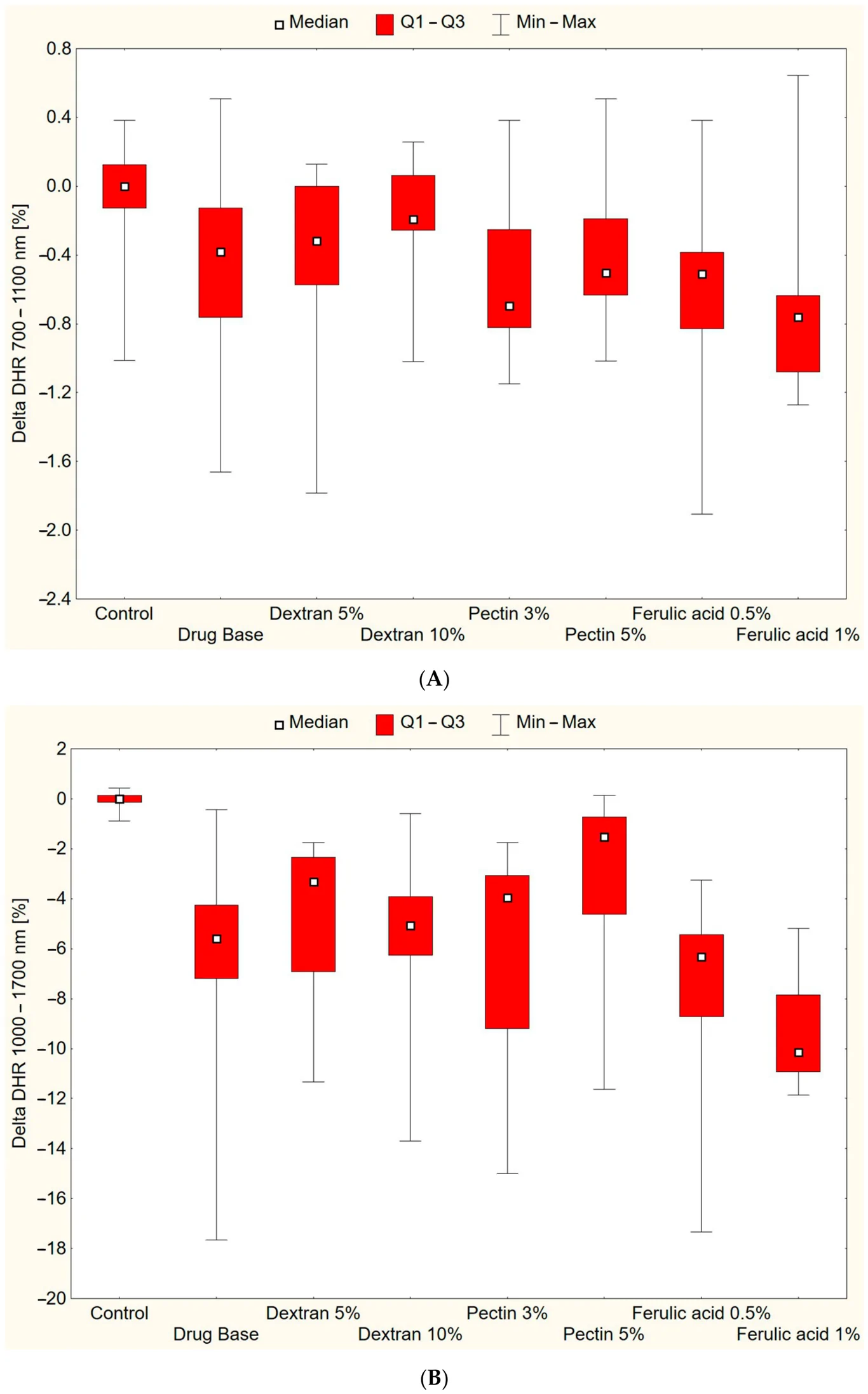

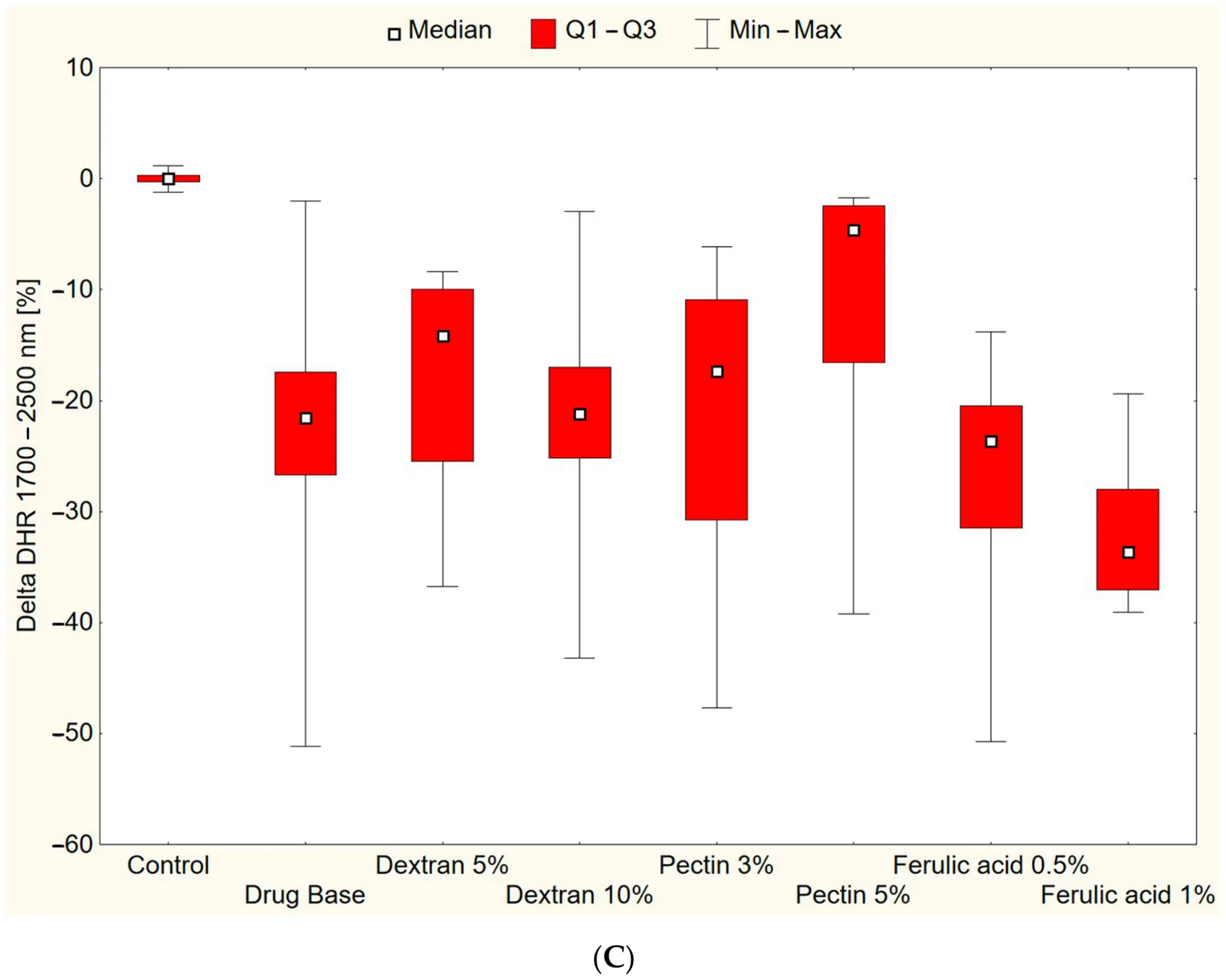
Percentage difference in directional-hemispheric reflectance (ΔDHR [%]) under model conditions (ex vivo) following application of the tested formulations, recorded in three spectral ranges of infrared radiation: (**A**) 700–1100 nm; (**B**) 1000–1700 nm; (**C**) 1700–2500 nm. Negative values on the Y-axis indicate a decrease in the reflectance coefficient, which directly correlates with an increase in radiation absorption by the applied formulation layer. The comparison visualizes the dominant effect of 1% ferulic acid on the drastic decrease in reflectance of the longest wavelengths (Panel (**C**)).

Statistical analysis of the differences in directional-hemispheric reflectance between the values obtained before and after application of the tested formulations in the spectral range of 700–1100 nm showed that the ΔDHR change for the test material differed significantly from the changes observed after application of the drug base (C vs. B; *p* < 0.001), 3% pectin (C vs. P3%; *p* = 0.001), 5% pectin (C vs. P5%; *p* = 0.004), and ferulic acid at concentrations of 0.5% and 1% (C vs. FA0.5% and C vs. FA1%, respectively; *p* < 0.001). However, no significant differences were found between ΔDHR in this regard for the drug base and the other tested formulations. The only exception was a statistically significant difference between the change induced by 1% ferulic acid and 10% dextran (FA1% vs. D10%; *p* = 0.009), as shown in Figure 1A.

In the 1000–1700 nm spectral range, the ΔDHR of the test material differed statistically significantly from the changes observed after the application of all tested preparations, i.e., the drug base, 5% and 10% dextran, 3% and 5% pectin, and 0.5% and 1% ferulic acid (*p* < 0.05). At the same time, no significant differences were observed between the changes seen for the drug base and the other formulations. Additionally, a statistically significant difference was found between the ΔDHR induced by 1% ferulic acid and the change following the application of 5% pectin (FA1% vs. P5%; *p* = 0.002), as shown in Figure 1B.

Similar relationships were observed in the 1700–2500 nm spectral range. The ΔDHR change for the test material was statistically significantly different compared to the drug base, 5% and 10% dextran, 3% and 5% pectin, and ferulic acid at both concentrations (*p* < 0.05). However, no significant differences were observed between the ΔDHR of the drug base and the changes caused by the other formulations. A statistically significant difference was again observed between 1% ferulic acid and 5% pectin (FA1% vs. P5%; *p* = 0.002), as shown in Figure 1C.

The results of the study showed that the directional thermal emissivity, measured at an incident angle of 20°, increased significantly after applying both the drug base alone and all the analyzed formulations—including 5% and 10% dextran, 3% and 5% pectin, and ferulic acid at concentrations of 0.5% and 1% (*p* < 0.001, Table 1). At the same time, a control measurement of the test material performed after the same elapsed time, without applying the preparation, showed no statistically significant changes compared to the initial measurement, confirming that time alone had no effect on the value of the tested parameter.

A similar trend was observed for measurements conducted at an incident angle of 60°. In this case as well, the application of the drug base and all tested formulations resulted in a statistically significant increase in directional thermal emissivity (*p* < 0.001), whereas a repeat measurement of the test material alone, performed after the same time interval as after the application of the formulations, revealed no significant differences from the initial values.

Analysis of the median percentage changes in thermal emissivity indicated that at an angle of 20°, these values were 0% for the test material, while after applying the formulations, an increase in emissivity was observed ranging from +0.9% for 5% pectin to +3.9% after applying 5% dextran. Lekobaza, 10% dextran, and ferulic acid at both concentrations caused a similar increase in emissivity, on the order of approximately +3%, while 3% pectin exhibited a moderate increase of +2.4%.

For an incident angle of 60°, the range of changes was more pronounced. The median percentage difference in directional thermal emissivity was 0% for the test material, while after application of the preparations, it ranged from +1.9% for 5% pectin to +5.3% for 5% dextran. 10% dextran and 3% pectin caused an increase in emissivity of approximately +5%, while the excipient and ferulic acid at both concentrations showed a moderate effect, with medians ranging from +2.7% to +3.5%.

**Figure 2:**
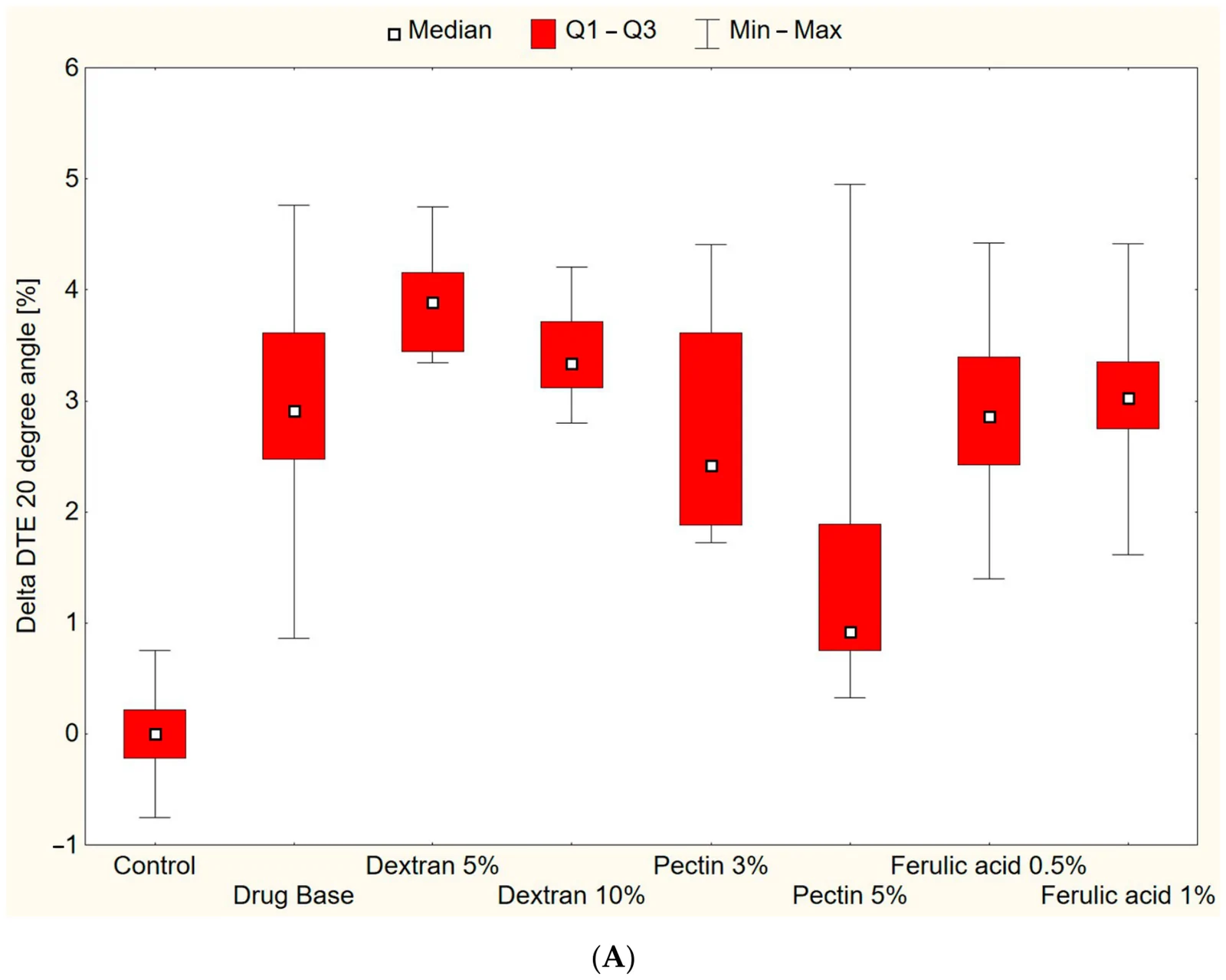

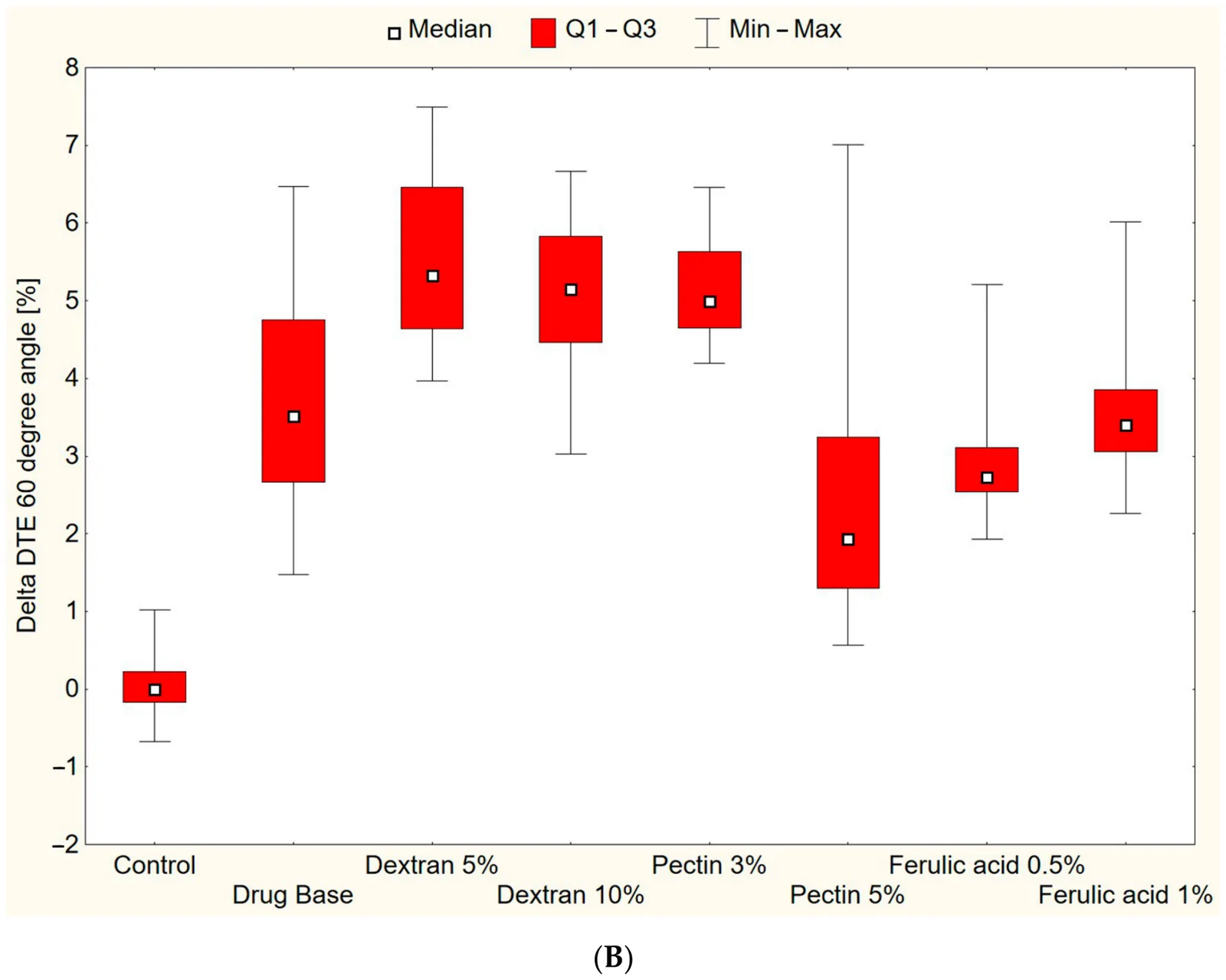
Percentage difference in directional thermal emissivity (ΔDTE [%]) in the 1.5–21 μm wavelength range under model conditions (ex vivo) following application of the tested formulations, measured at radiation incidence angles of: (**A**) 20° and (**B**) 60°. Positive values indicate a physical increase in the system’s emissivity relative to the pre-application state.

As shown in Figure 2A, a detailed statistical analysis of the differences between the values obtained before and after application of the formulations revealed that the change in directional thermal emissivity at a 20° angle for the test material differed statistically significantly from the changes observed after application of the drug base, 5% and 10% dextran, 3% pectin, and ferulic acid at both concentrations (*p* < 0.001). At the same time, no significant difference was found between the change in emissivity for the test material and the effect induced by 5% pectin, indicating a relatively small influence of this formulation at a 20° angle. No statistically significant differences were observed between the vehicle and the other tested formulations. Significant differences, however, were found in comparisons between 5% pectin and 5% and 10% dextran, with 5% pectin causing a significantly smaller increase in emissivity.

With regard to the measurements taken at an incident angle of 60°, shown in Figure 2B, it was found that the change in directional thermal emissivity for the test material was statistically significantly different compared to all tested formulations, including the drug base, dextrans, pectins, and ferulic acid (*p* < 0.05). As with the 20° angle, no significant differences were observed between the drug base and the other formulations. However, a detailed comparison analysis revealed statistically significant differences between 5% and 10% dextrans and 5% pectin, as well as between dextrans and 0.5% ferulic acid, with dextrans causing a greater increase in emissivity. Furthermore, 3% pectin exhibited a significantly greater effect than 5% pectin and 0.5% ferulic acid.

## 3. Discussion

Studies conducted to evaluate the photoprotective potential of selected cosmetic ingredients—ferulic acid, pectin, and dextran—in the infrared (IR) radiation range using the directional hemispheric reflectance (DHR) method have provided significant data allowing for the verification of existing views on photoprotection mechanisms in the long-wavelength range. The key and most surprising finding of this study is the observation that the application of the tested formulations to the surface of an ex vivo model led, in most cases, to a statistically significant reduction in the reflectance coefficient compared to the uncoated surface. This result appears to contradict the classical definition of a physical sunscreen, whose purpose is to maximize the scattering and reflection of radiation in order to limit its transmission to deeper layers of the skin [10,17]. To properly interpret these results, it is necessary to refer to the fundamental laws of tissue optics, thermodynamics, and the specific spectral characteristics of the raw materials used, particularly in the context of the dominant role of water in cosmetic formulations [18].

First, we must address the observed phenomenon of a decrease in reflectance following the application of the base (Lekobaza) and active formulations. In the literature on this subject, including the works of Wilczyński et al., it has been repeatedly emphasized that the measurement of skin reflectance or skin model reflectance is the result of two phenomena: specular reflection from the surface of the stratum corneum (or the surface of the material) and diffuse reflection resulting from multiple scattering of photons within the material’s structure. Dry skin (or a model material) is characterized by a significant difference in refractive indices at the air-material interface (n_(air) = 1.0 vs. n_(skin) = 1.55). This difference, according to Fresnel’s equations, generates specular reflection. The application of a cosmetic preparation—an emulsion or hydrogel with a refractive index close to 1.33–1.40—introduces an intermediate layer on the surface that optically matches the refractive indices. This phenomenon, known in optics as index matching, leads to a reduction in surface reflection and facilitates the penetration of photons into the material. The obtained results, indicating a decrease in reflectance in the 700–1100 nm range (near-infrared, IR-A) after the application of Lekobaza and formulations containing pectin or dextran, confirm that in this spectral range the tested preparations do not act as scattering screens (such as titanium dioxide), but rather increase the optical transparency of the surface to IR radiation. This is a key finding for the design of photoprotection: the mere application of a layer of cosmetic, even one containing active ingredients, may paradoxically increase the energy dose deposited in the skin if these ingredients lack the ability to strongly backscatter light [10,17].

Particular attention should be paid to the results obtained for ferulic acid. This substance is widely recognized as the gold standard in antioxidant protection against UV radiation, due to its ability to stabilize vitamins C and E and its direct absorption of UVB/UVA. However, this study demonstrated that 1% ferulic acid caused the greatest decrease in reflectance in the 1700–2500 nm range (reaching a median of 33.7%). Interpretation of this phenomenon requires analysis of the near-infrared (NIR) absorption spectrum. In contrast to the UV range, where electronic transitions dominate, absorption in the NIR range results from overtones and vibrational combination bands of chemical bonds, primarily O-H, C-H, and N-H. Ferulic acid, as a structure rich in hydroxyl groups (phenolic and carboxylic) and C-H bonds in the aromatic ring and side chain, exhibits a series of absorption bands in the range above 1400 nm. A sharp decrease in reflectance (indicating increased absorption by the sample layer) suggests that ferulic acid acts as an energy absorber in this spectral range [18,19]. From a thermodynamic perspective, the absorbed IR radiation energy does not disappear but is dissipated as heat. This means that a high concentration of ferulic acid on the skin surface may lead to a local increase in epidermal temperature under the influence of sun exposure. In the context of research on thermal aging, where elevated temperature stimulates angiogenesis and induces MMP-1 expression independently of free-radical pathways, this property can be viewed as ambivalent. On the one hand, ferulic acid neutralizes radicals formed in the mitochondria (biological mechanism); on the other hand, however, as a physical absorber in the IR-B band, it may contribute to thermal stress on the tissue [4,6,20,21].

An analysis of the effects of biopolymers—pectin and dextran—sheds light on the role of hydration in interactions with infrared radiation. Pectin and dextran are hydrocolloids with strong hygroscopic properties. When incorporated into an emulsion base (Lekobaza), they form a water-binding film on the skin’s surface. Water is a key chromophore in the infrared range, exhibiting intense absorption bands around 1450 nm (the first overtone of O-H stretching vibrations) and 1940 nm (the combination band of stretching and bending vibrations). These results, showing a drastic decrease in reflectance in the 1700–2500 nm range for all tested samples, correlate perfectly with the presence of the water absorption band at 1940 nm. It can be hypothesized that the observed “radiation-protective” effect in the sense of reduced transmission (assuming that the decrease in reflectance is due to absorption within the cosmetic layer rather than transmission to the dermis) is in fact a “water-filtering” effect. A layer of a water-rich formulation acts as a filter blocking IR-B and IR-C radiation. Although this mechanism protects the deeper layers of the dermis from direct IR exposure, it comes at the cost of heating the stratum corneum itself and the epidermal surface, where energy is deposited [10,18]. In the dermatological literature [11]., there is debate over whether such superficial absorption is beneficial. Retaining IR on the surface protects fibroblast mitochondria but induces thermal stress in keratinocytes and may lead to the formation of erythema ab igne-type lesions. The variation in results between pectin and dextran, as well as the concentration-dependent nature (e.g., differences between D5% and D10%), suggest the possibility of modulating this effect by altering the structure of the formed film—more dense polymer cross-linking may influence the amount of bound water and the scattering phenomena at the polymer/air phase boundary within the film’s micropores [11]. A simple order-of-magnitude estimate supports this interpretation: using the optical constants of water [18], the 1/e absorption depth in the 1900–2000 nm band is of the order of 10^2^ µm, so that for a ~20 µm film measured against a reflective substrate (i.e., a double optical path) the expected single-layer absorptance is roughly 15–30%. This is of the same order as the measured reduction in directional-hemispheric reflectance in the 1700–2500 nm band (median ΔDHR up to −33.7% for 1% ferulic acid), indicating that the observed decrease reflects genuine absorption of near-infrared radiation within the hydrated film rather than a measurement artifact.

An important element is also the analysis of thermal emissivity. The increase in emissivity observed after the application of all formulations (at both 20° and 60° angles) demonstrates that applying a layer of the cosmetic changes the surface’s radiative characteristics toward those of a blackbody (emissivity close to 1). According to Kirchhoff’s law, high emissivity in a given wavelength range also implies a high capacity to absorb thermal radiation from the environment. Increased emissivity promotes more efficient radiation of metabolic and absorbed heat (radiative cooling), which is a desirable phenomenon for lowering skin temperature. However, under conditions of strong sunlight, the energy balance is dominated by the absorption of short-wave IR (solar), and high emissivity mainly concerns the far-infrared (FIR, >3000 nm) range. These results suggest that the tested formulations may support the skin’s thermoregulatory mechanisms by facilitating heat emission, which constitutes a potential physical protective mechanism against thermal aging, independent of optical barrier properties [6,20].

When comparing the obtained results with those reported by other authors, it is worth noting the consistency with the findings from studies on traditional UV filters. The work of Stolecka-Warzecha et al. and Wilczyński demonstrated that classic organic and mineral filters (in their standard micronized forms) are largely transparent to IR radiation. This study extends this knowledge to a group of natural compounds (polysaccharides, phenolic acids). It has been demonstrated that, like synthetic filters, the natural substances under investigation do not possess the ability to broadband-reflect IR in their native form (dissolved or gel-like). This fundamentally distinguishes them from materials used in protective clothing or materials engineering, where photonic structures or special pigments (e.g., TiO_2_ with large particle diameters > 400 nm) are specifically designed to scatter infrared radiation. The lack of a significant increase in reflectance in the tested samples suggests that in cosmetology “IR protection” based on these ingredients must be defined rather as biochemical protection (antioxidation, quenching of excited states) or thermoregulatory protection (increased emissivity, evaporation of water from the gel matrix), rather than as physical shielding (a shield) [10,17].

It is also worth raising the issue of the limitations of an ex vivo model based on synthetic material in the context of extrapolating results to human skin in vivo. Although the model used ensures surface standardization and measurement reproducibility (as confirmed by low standard deviations), it does not account for dynamic physiological processes such as sweating, sebum secretion, or microcirculation, which can modify both the hydration of the stratum corneum and surface temperature. Nevertheless, isolating the physical aspect of radiation interaction with the cosmetic layer is an advantage of this model, as it allows the observed changes to be unambiguously attributed to the properties of the formulation itself, without interference from individual variability [6,22]. It should also be noted that the individual DHR values showed a wider spread in the 1700–2500 nm band than the uncoated control (which varied by less than 2%); this reflects the intrinsically low absolute reflectance of the water-rich layer in this strongly water-absorbing region (around 1940 nm), where small absolute differences translate into large relative variations, together with unavoidable replicate-to-replicate differences in the thickness and hydration of the manually applied film. Two further methodological limitations should be acknowledged. First, because the ET-100 is a contact instrument and the formulations are soft, a minor and unavoidable degree of surface conformation of the film beneath the measuring window cannot be entirely excluded; the stability of the uncoated control fields (variation < 2%) indicates, however, that this does not introduce a systematic artifact. Second, the present optical configuration characterizes the radiation returned from, and emitted by, the coated surface, but does not directly measure the absolute fraction of radiation transmitted through the film to a simulated dermis; a strict quantification of shielding would require a dedicated transmission measurement (for example, a detector placed behind a thin skin-mimicking absorber, or the spectral transmittance of free-standing films). Accordingly, the present data should be interpreted as characterizing the modulation of the surface’s radiative interaction with IR—a redistribution of incident energy through enhanced absorption in the outer layer and enhanced re-emission—rather than as an absolute measurement of transmitted (blocked) radiation.

In summary, the study results indicate the complex nature of “radiation-protective potential” in the infrared spectrum. This term should not be equated solely with reflectance (as in the case of SPF). Ferulic acid, although it causes a decrease in reflectance (increase in absorption), remains a valuable ingredient due to its proven ability in other studies to neutralize free radicals generated by this absorption. In turn, polysaccharides (pectin, dextran) modify the absorption profile mainly through interaction with water, creating an absorption barrier for the IR-B and IR-C bands, which may reduce radiation penetration into the dermis but increase the thermal load on the epidermis. These findings imply the need to change the approach to the formulation of IR-protection products. Effective protection should combine three pillars: (1) physical reflection (reflectance)—which would require adding scattering agents with appropriate particle size to the tested bases, (2) surface absorption combined with a cooling system (e.g., water evaporation from polysaccharide hydrogels), and (3) neutralization of biological effects (antioxidants such as ferulic acid). This study demonstrates that none of the tested ingredients, when used as monotherapy, provides full physical protection (high reflectance); however, their properties may be complementary in complex formulation systems. Further research should focus on evaluating how the modification of emissivity and absorption by these ingredients translates into actual changes in tissue temperature in vivo and the expression of heat stress markers [5,6,10,19].

## 4. Materials and Methods

### 4.1. Physical Characteristics of the Components

Directional-hemispheric reflectance measurements were used to analyze, among others, dextran, citrus pectin, and ferulic acid, evaluating their properties and potential radioprotective effects in the infrared radiation range.

Dextran from *Leuconostoc* spp. (Mr 40,000; product No. 10278392; CAS No. 9004-54-0), pectin from citrus peel with a galacturonic acid content ≥ 74.0% (product No. 0000311421; CAS No. 9000-69-5), and trans-ferulic acid ≥ 99% (product No. 10271448; CAS No. 537-98-4) were purchased from Sigma-Aldrich (St. Louis, MO, USA).Dextran is a polysaccharide produced by microorganisms, composed of glucose residues linked primarily by α-1,6-glycosidic bonds, with a small proportion of branches formed by α-1,4, α-1,3, and α-1,2 bonds [14].

Pectin is a natural plant polysaccharide with a chain composed mainly of D-galacturonan molecules (α-1,4 glycosidic bonds). It is found in plant cell walls and is widely used in industry as a gelling, thickening, stabilizing, and emulsifying agent. Pectin can have varying degrees of methylation, which affects how it forms gels or modifies the consistency of food or cosmetic products [15,16].

Ferulic acid (4-hydroxy-3-methoxycinnamic acid) is a natural phenolic hydroxycinnamic compound widely found in plants, especially in grains, fruits, and vegetables, usually in a form bound to cell wall polysaccharides. Chemically, it consists of a phenolic ring with hydroxyl and methoxyl groups and a side chain of α,β-unsaturated carboxylic acid, which gives it strong antioxidant and free-radical scavenging properties. This structure allows for the stabilization of phenoxy radicals through resonance, which contributes to its effective protection of cells against oxidative stress. Ferulic acid also exhibits anti-inflammatory and antimicrobial effects, as well as potential health benefits in the context of metabolic diseases such as insulin resistance and dyslipidemia. Furthermore, it is used in the food industry as a natural preservative and antioxidant, as well as in cosmetology, including in anti-aging and photoprotective products [18,19].

### 4.2. Formulation of Preparations and Evaluation of Their Organoleptic Properties

Formulations containing 5% and 10% dextran, 3% and 5% pectin, and 0.5% and 1% ferulic acid were developed. Lekobaza Pharma Cosmetic (Fagron a.s., Holicka 1098/31m, 779 00 Olomouc, Czech Republic), a pharmaceutical raw material from batch 111678 with an expiration date of 30 June 2027, was used as the ointment base.

Lekobaza is an amphiphilic base containing O/W (polysorbate 40) and W/O (glycerol monostearate and cetostearyl alcohol) emulsifiers, which allows for the absorption of both the aqueous and oil phases. It is characterized by a pH of 5.5, a soft consistency, good spreadability, ease of removal, and high skin tolerance. The formulations were prepared by weighing the appropriate amount of the ingredient, adding it to the base, and thoroughly mixing it using a formulation mixer [23].

**Figure 3:**
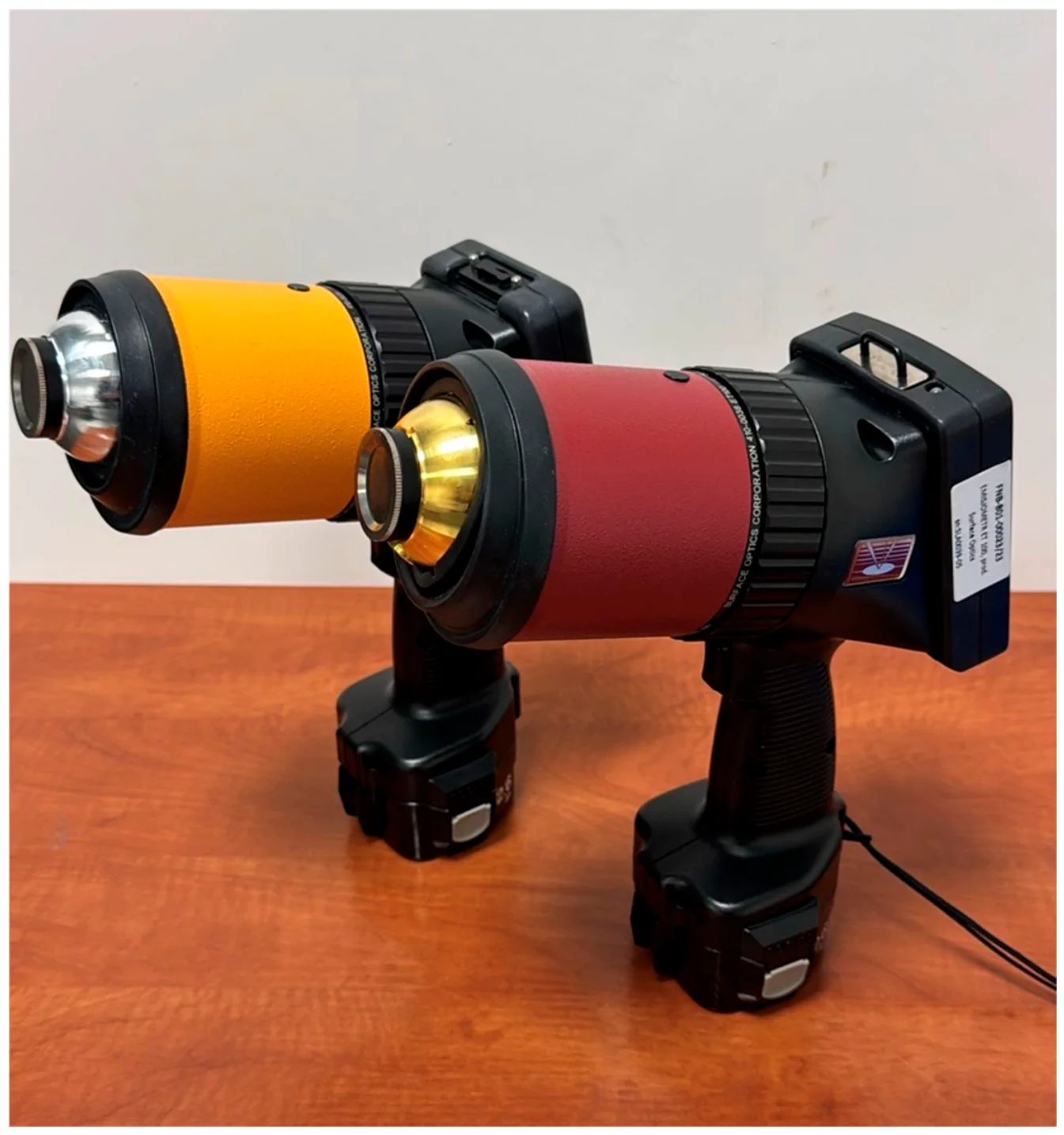
Reflectometer Solar 410 and Emissometer ET-100 (Surface Optics Corporation).

In laboratory studies, a 1 mm-thick synthetic silicone material was used as the application surface to evaluate the photoprotective properties of the analyzed ingredients. The formulations listed in Table S1 and the base material, which served as the test substrate for sample preparation, were analyzed. Directional-hemispheric reflectance measurements were performed using a Solar 410 reflectometer (Surface Optics Corporation, San Diego, CA, USA), while emissivity was determined using an ET-100 emissometer (Surface Optics Corporation, USA) (Figure 3). The SOC 410-Solar reflectometer is an integrating-sphere instrument that measures total, diffuse and specular directional–hemispherical reflectance at near-normal (20°) incidence, averaged within seven spectral bands between 335 and 2500 nm (335–380, 400–540, 480–600, 590–720, 700–1100, 1000–1700 and 1700–2500 nm), in accordance with ASTM E903 and ASTM C1549. The SOC ET-100 emissometer records in-band directional reflectance over six bands spanning 1.5–21 µm (1.5–2.0, 2.0–3.5, 3.0–4.0, 4.0–5.0, 5.0–10.5 and 10.5–21 µm) at incidence angles of 20° and 60°, and derives directional and total hemispherical emissivity from the relationship ε = 1 − ρ, in accordance with ASTM E408; according to the manufacturer, its emittance accuracy is ±0.03 and its repeatability ±0.005. Both instruments were calibrated before each measurement session against the manufacturer’s high- and low-reflectance (and high-/low-emittance) reference standards. Measurement fields measuring 3 cm × 3 cm were defined on the surface of the test material. The first measurement was performed before applying the sample. Next, a formulation or drug base was weighed onto a spatula in an amount corresponding to 2 mg/cm^2^ (average mass 20 mg ± 1.7 mg), applied to the designated area, and reflectance and emissivity measurements were taken. Additionally, control measurements of the test material were performed after a period of time corresponding to the time elapsed since the application of the sample. The entire procedure was repeated 16 independent replicate measurements (*n* = 16), rather than as a kinetic time series, for each of the tested formulations. All measurements were carried out under constant ambient laboratory conditions (air temperature 22 ± 1 °C, relative humidity 45 ± 5%), so that the temperature of the silicone substrate remained constant throughout each measurement series. For every replicate, the “after” reading was recorded immediately following application of the formulation. Because the ET-100 emissometer measures reflectance—a temperature-independent physical quantity—and derives emissivity from the relationship ε = 1 − ρ under the assumption that the sample is opaque (negligible transmission) in the 1.5–21 µm range, the reported emissivity values are not affected by minor fluctuations in sample temperature. To ensure a valid contact emissivity reading on these soft samples, the ET-100 window was seated perpendicularly against the film-on-substrate stack, so that the contact force was borne by the rigid 1 mm silicone support rather than by the film alone, and the substrate defined a fixed measurement plane. Each reading consisted of a single, brief (<10 s) and light placement of the window; the instrument was neither pressed into, slid across, nor repeatedly applied to the same film, and every one of the sixteen replicates was performed on a freshly applied film. Because the optically relevant quantity is the areal mass density (2 mg/cm^2^) and the composition of the material within the aperture, a slight local redistribution of the essentially incompressible, water-rich film under gentle normal contact does not alter the quantity interrogated by the instrument. Each sample was equilibrated to ambient temperature before measurement; within the ± 1 °C window the associated change in film viscosity is negligible and affects neither the areal density nor the infrared absorption of the layer, while the brief contact with the room-temperature window induces no measurable change in sample temperature. The opacity assumption (τ = 0, ε = 1 − ρ) applies specifically to the derivation of thermal emissivity in the 1.5–21 µm band, where a water-rich film on a silicone substrate is effectively opaque, and is not applied to the near-infrared directional-hemispheric reflectance measurements.

### 4.3. Statistical Analysis

The obtained data were initially processed using Microsoft^®^ Excel^®^ 2019 MSO, version 2503 (Microsoft Corporation, Redmond, WA, USA). Statistical analyses were performed using Statistica 13.3 software (TIBCO Software Inc., Palo Alto, CA, USA). Statistical significance was set at *p* < 0.05. The normality of data distribution was assessed using the Shapiro–Wilk test and visual inspection of histograms. Since some variables did not meet the assumption of normality, non-parametric statistical tests were applied.

## 5. Conclusions

This ex vivo study demonstrates that ferulic acid, pectin, and dextran modulate the interaction of infrared radiation with a surface not through specular reflection or backscattering, but through a significant change in the kinetics of energy absorption and emission.

Ferulic acid exhibited the highest absorption potential, manifested by a statistically significant decrease in directional-hemispheric reflectance, which, combined with its documented antioxidant activity, positions it as a multifunctional agent protecting against the effects of photochemical and thermal stress.

In turn, polysaccharide biopolymers, by forming a hydrocolloid matrix on the surface, act as selective water filters that, through the absorption of long-wavelength radiation in the outer layer, reduce the transmission of radiation to deeper structures of the dermis.

A significant discovery with practical implications is the ability of all tested formulations to increase thermal emissivity, which promotes more effective dissipation of absorbed energy through radiative cooling and may support the tissue’s natural thermoregulation. This indicates that the concept of IR photoprotection should evolve from simple shielding toward active management of the heat balance to prevent thermal aging-related changes.

The methodology used to measure optical parameters on a synthetic substrate serves as a reliable preformulation tool, allowing for the optimization of a product’s thermodynamic properties at an early stage of its development, while eliminating individual variability. Ultimately, these results justify the design of hybrid protective systems that integrate physical energy absorption with thermoregulatory mechanisms, offering comprehensive protection against a wide spectrum of environmental factors.

## Figures and Tables

**Table 1:** Directional thermal emissivity before and after application of the tested formulations at incident angles of 20° and 60°, under model conditions (ex vivo).

	Sample	Measurement	Me	Q1	Q3	Min	Max	Avg	SD	*p*
DTE 20° [a.u.]	C	Before	0.930	0.929	0.931	0.925	0.935	0.930	0.002	0.635
		After	0.931	0.929	0.932	0.923	0.935	0.930	0.002	
	B	Before	0.928	0.927	0.930	0.924	0.933	0.928	0.002	<0.001
		After	0.956	0.951	0.962	0.935	0.970	0.956	0.008	
	D5%	Before	0.928	0.927	0.928	0.925	0.930	0.927	0.001	<0.001
		After	0.963	0.961	0.966	0.959	0.971	0.964	0.003	
	D10%	Before	0.928	0.928	0.930	0.926	0.930	0.928	0.001	<0.001
		After	0.959	0.957	0.962	0.955	0.967	0.960	0.004	
	P3%	Before	0.929	0.928	0.930	0.926	0.933	0.929	0.002	<0.001
		After	0.952	0.947	0.962	0.944	0.971	0.955	0.009	
	P5%	Before	0.929	0.928	0.929	0.926	0.930	0.928	0.001	<0.001
		After	0.938	0.936	0.947	0.930	0.975	0.942	0.012	
	FA0.5%	Before	0.928	0.927	0.929	0.926	0.930	0.928	0.001	<0.001
		After	0.954	0.951	0.959	0.943	0.968	0.955	0.007	
	FA1%	Before	0.927	0.926	0.928	0.925	0.929	0.927	0.001	<0.001
		After	0.955	0.953	0.959	0.944	0.969	0.956	0.006	
DTE 60° [a.u.]	C	Before	0.885	0.883	0.886	0.878	0.889	0.884	0.002	0.306
		After	0.885	0.883	0.886	0.877	0.893	0.885	0.003	
	B	Before	0.883	0.882	0.885	0.877	0.889	0.883	0.002	<0.001
		After	0.916	0.907	0.925	0.895	0.938	0.916	0.010	
	D5%	Before	0.884	0.883	0.885	0.880	0.889	0.884	0.002	<0.001
		After	0.932	0.926	0.941	0.918	0.947	0.932	0.009	
	D10%	Before	0.885	0.884	0.887	0.882	0.892	0.886	0.002	<0.001
		After	0.931	0.926	0.936	0.918	0.944	0.931	0.008	
	P3%	Before	0.881	0.879	0.882	0.877	0.884	0.881	0.002	<0.001
		After	0.924	0.923	0.929	0.917	0.940	0.926	0.006	
	P5%	Before	0.882	0.880	0.884	0.878	0.889	0.882	0.003	<0.001
		After	0.900	0.894	0.909	0.891	0.947	0.904	0.014	
	FA0.5%	Before	0.883	0.882	0.884	0.879	0.887	0.883	0.002	<0.001
		After	0.908	0.903	0.913	0.900	0.929	0.909	0.008	
	FA1%	Before	0.884	0.883	0.884	0.880	0.892	0.884	0.003	<0.001
		After	0.914	0.912	0.918	0.904	0.934	0.915	0.007	

DTE 20°—directional thermal emissivity at an incident angle of 20°; a.u.—arbitrary units; DTE 60°—directional thermal emissivity at an incident angle of 60°; a.u.—arbitrary units; C—test material; B—material with a leko base; D5%—material with 5% dextran; D10%—material with 10% dextran; P3%—material with 3% pectin; P5%—material with 5% pectin; FA0.5%—material with 0.5% ferulic acid; FA1%—material containing 1% ferulic acid; Me—median; Q1—first quartile (25%); Q3—third quartile (75%); Min—minimum; Max—maximum; Avg—average; SD—standard deviation; *p*—level of statistical significance.

## Data Availability

The data presented in this study are available on request from the corresponding author. The data are not publicly available due to the sensitive nature of the data.

## Supplementary Materials

The following supporting information can be downloaded at: https://www.mdpi.com/article/10.3390/ph19081143/s1, Table S1: Characteristics of the formulations studied; Table S2: Directional-hemispheric reflectance before and after application of the tested formulations across all tested spectral ranges, under model conditions (ex vivo).
