# Supplementary material for: Evaluation of Infrared Photoprotective Potential of Cosmetic Ingredients Using Directional-Hemispherical Reflectance in an Ex Vivo Model
Source: Pharmaceuticals (Basel). 2026 Jul 24;19(8):1143. doi: 10.3390/ph19081143 (PMC13516776; doi:10.3390/ph19081143)
Supplement: Supplementary file 1 [file pharmaceuticals-19-01143-s001.zip › pharmaceuticals-4372423-supplementary.pdf]

Supplementary Materials

Title: Evaluation of Infrared Photoprotective Potential of Cosmetic Ingredients using Directional-Hemispherical Reflectance in an Ex Vivo Model

Authors: Elżbieta Mickoś . Paula Babczyńska. Sławomir Wilczyński and Magdalena Hartman-Petrycka

Journal: Pharmaceuticals

Supplementary Tables

Table S1. Characteristics of the formulations studied

Ingredient name	Sample identification	Concentration of the ingredient	Organoleptic evaluation
Dextran (Dextran z Leuconostoc spp. Mr 40 000)	D5%	5%	a homogeneous. smooth. semi-solid mass with no visible lumps; uniform white color; soft consistency at a 5% concentration. easy to spread. and thick at a 10% concentration. requiring thorough application; odor typical of the Lekobaza base.
	D10%	10%	
Pectin (Pectin from citrus peel. galacturonic acid ≥ 74.0%)	P3%	3%	a homogeneous. smooth. semi-solid form. free of lumps; an off-white. uniform color; a thick consistency for both concentrations (3% and 5%). requiring thorough spreading; an odor characteristic of the base.
	P5%	5%	
Ferulic acid (Trans-Feluric acid 99%)	FA0.5%%	0.5%	a homogeneous. smooth. semi-solid mass free of lumps; a uniform creamy white color; a soft consistency at concentrations of 0.5% and 1%. easy to spread on the skin; an odor characteristic of the base
	FA0.5%	1%	

Table S2. Directional-hemispheric reflectance before and after application of the tested formulations across all tested spectral ranges. under model conditions (ex vivo)

	Sample	Measurement	Me	Q1	Q3	Min	Maks	Avg	SD	p
DHR λ 700–1100 nm [a.u.]	C	Before	0.788	0.786	0.790	0.781	0.793	0.788	0.002	0.059
		After	0.787	0.785	0.789	0.781	0.794	0.787	0.003	
	B	Before	0.786	0.785	0.788	0.780	0.795	0.787	0.003	0.000
		After	0.783	0.781	0.785	0.770	0.791	0.783	0.004	
	D5%	Before	0.785	0.784	0.785	0.782	0.786	0.785	0.001	0.004
		After	0.782	0.780	0.784	0.770	0.786	0.782	0.004	
	D10%	Before	0.784	0.783	0.785	0.781	0.787	0.784	0.002	0.083
		After	0.783	0.782	0.784	0.776	0.786	0.783	0.003	
	P3%	Before	0.789	0.787	0.790	0.784	0.794	0.789	0.003	0.002
		After	0.786	0.783	0.787	0.775	0.789	0.785	0.004	
	P5%	Before	0.791	0.790	0.793	0.786	0.794	0.791	0.002	0.002
		After	0.789	0.785	0.790	0.779	0.790	0.787	0.003	
	FA0.5%	Before	0.785	0.782	0.787	0.779	0.787	0.784	0.002	<0.001
		After	0.780	0.778	0.782	0.772	0.785	0.779	0.003	
	FA1%	Before	0.786	0.785	0.788	0.777	0.789	0.786	0.003	<0.001
		After	0.780	0.779	0.782	0.777	0.783	0.780	0.002	
DHR λ 1000–1700 nm [a.u.]	C	Before	0.681	0.680	0.683	0.675	0.686	0.681	0.002	0.172
		After	0.681	0.679	0.683	0.676	0.686	0.681	0.002	
	B	Before	0.681	0.680	0.683	0.674	0.690	0.681	0.003	<0.001
		After	0.643	0.633	0.653	0.555	0.681	0.641	0.019	
	D5%	Before	0.680	0.679	0.681	0.678	0.681	0.680	0.001	<0.001
		After	0.657	0.632	0.664	0.602	0.669	0.648	0.020	
	D10%	Before	0.679	0.678	0.680	0.676	0.682	0.679	0.002	<0.001
		After	0.646	0.637	0.652	0.586	0.674	0.645	0.020	
	P3%	Before	0.683	0.681	0.684	0.678	0.688	0.683	0.002	<0.001
		After	0.656	0.619	0.663	0.578	0.671	0.641	0.030	
	P5%	Before	0.686	0.684	0.687	0.680	0.688	0.685	0.002	0.001
		After	0.677	0.652	0.681	0.601	0.683	0.664	0.027	
	FA0.5%	Before	0.679	0.677	0.681	0.674	0.682	0.679	0.002	<0.001
		After	0.636	0.619	0.642	0.558	0.656	0.627	0.026	
	FA1%	Before	0.681	0.680	0.683	0.674	0.683	0.681	0.002	<0.001
		After	0.612	0.608	0.628	0.602	0.644	0.617	0.014	
DHR λ 1700–2500 nm [a.u.]	C	Before	0.340	0.339	0.342	0.335	0.345	0.340	0.002	0.304
		After	0.340	0.339	0.342	0.335	0.347	0.340	0.002	
	B	Before	0.342	0.340	0.344	0.335	0.348	0.342	0.003	<0.001
		After	0.270	0.250	0.284	0.164	0.339	0.267	0.027	
	D5%	Before	0.342	0.341	0.343	0.337	0.345	0.342	0.002	<0.001
		After	0.293	0.255	0.308	0.213	0.316	0.281	0.032	
	D10%	Before	0.341	0.340	0.342	0.338	0.344	0.341	0.002	<0.001
		After	0.270	0.255	0.284	0.192	0.331	0.273	0.034	
	P3%	Before	0.343	0.343	0.344	0.338	0.346	0.343	0.002	<0.001
		After	0.283	0.236	0.306	0.179	0.323	0.269	0.046	
	P5%	Before	0.345	0.344	0.346	0.340	0.347	0.345	0.002	<0.001
		After	0.331	0.287	0.337	0.209	0.340	0.308	0.044	
	FA0.5%	Before	0.340	0.339	0.342	0.337	0.345	0.340	0.002	<0.001

	After	0.261	0.233	0.271	0.166	0.294	0.250	0.034	
FA1%	Before	0.343	0.342	0.345	0.338	0.348	0.343	0.002	<0.001
	After	0.226	0.218	0.247	0.209	0.275	0.233	0.021	

DHR λ 700–1100 nm – directional hemispheric reflectance in the spectral range of 700–1100 nm; DHR λ 1000–1700 nm – directional hemispheric reflectance in the spectral range of 1000–1700 nm; DHR λ 1700–2500 nm – directional hemispherical reflectance in the spectral range of 1700–2500 nm; a.u. – arbitrary units; C – test material; B – material with a drug base; D5% – material with 5% dextran; D10% – material with 10% dextran; P3% – material with 3% pectin; P5% – material with 5% pectin; FA0.5% – material with 0.5% ferulic acid; FA1% – material with 1% ferulic acid; Me – median; Q1 – first quartile (25%); Q3 – third quartile (75%); Min – minimum; Max – maximum; Avg – average; SD – standard deviation; p – level of statistical significance
